# Another Specific

**Published:** 1886-02

**Authors:** 


### ANOTHER SPECIFIC.

Dr. Darling, in the *Therapeutic Gazette*, says that half a drachm of muriate of ammonia is the best remedy in neuralgia and toothache. That is quite as definite as to say that quinine is an excellent remedy for illness.

